# Exploring the Spatial and Temporal Patterns of Children and Adolescents with COVID-19 Infections in Slovakia during March 2020 to July 2022

**DOI:** 10.3390/medicina60060931

**Published:** 2024-06-02

**Authors:** Ahmad Gharaibeh, Mamoun A. Gharaibeh, Siham Bataineh, Anna Maria Kecerová

**Affiliations:** 1 Teaching Department of Orthopaedics Musculoskeletal Trauma, Faculty of Medicine, University Hospital of Louise Pasteur, Pavel Jozef Safarik University, 040 11 Košice, Slovakia; 2 Department of Natural Resources and the Environment, Faculty of Agriculture, Jordan University of Science and Technology, P.O. Box 3030, Irbid 22110, Jordan; 3 Department of Civil Engineering, Faculty of Engineering, Jordan University of Science and Technology, P.O. Box 3030, Irbid 22110, Jordan; seham80@just.edu.jo; 4 Social Insurance Headquarter, 040 84 Košice, Slovakia; erikdorco@pobox.sk

**Keywords:** COVID-19, spatial patterns, Slovakia, age group

## Abstract

*Background and Objectives*: The COVID-19 pandemic has had a significant global impact, necessitating a comprehensive understanding of its spatiotemporal patterns. The objective of this study is to explore the spatial and temporal patterns of COVID-19 infections among five age groups (<1, 1–4, 5–9, 10–14, and 15–19 years) in 72 districts of Slovakia on a quarterly basis from March 2020 to July 2022. *Material and Methods*: During the study period, a total of 393,429 confirmed PCR cases of COVID-19 or positive antigen tests were recorded across all studied age groups. The analysis examined the spatiotemporal spread of COVID infections per quarter, from September 2021 to May 2022. Additionally, data on hospitalizations, intensive care unit (ICU) admissions, pulmonary ventilation (PV), and death cases were analyzed. *Results*: The highest number of COVID-19 infections occurred between September 2021 and May 2022, particularly in the 10–14-year-old group (68,695 cases), followed by the 15–19-year-old group (62,232 cases), while the lowest incidence was observed in the <1-year-old group (1235 cases). Out of the total confirmed PCR cases, 18,886 individuals required hospitalization, 456 needed ICU admission, 402 received pulmonary ventilation, and only 16 died. The analysis of total daily confirmed PCR cases for all regions showed two major peaks on 12 December 2021 (6114 cases) and 1 February 2022 (3889 cases). Spatial mapping revealed that during December 2021 to February 2022, the highest number of infections in all age groups were concentrated mainly in Bratislava. Moreover, temporal trends of infections within each age group, considering monthly and yearly variations, exhibited distinct spatial patterns, indicating localized outbreaks in specific regions. *Conclusions*: The spatial and temporal patterns of COVID-19 infections among different age groups in Slovakia showed a higher number of infections in the 10–14-year-old age group, mainly occurring in urban districts. The temporal pattern of the spread of the virus to neighboring urban and rural districts reflected the movement of infected individuals. Hospitalizations, ICU and PV admissions, and deaths were relatively low. The study highlights the need for more proactive measures to contain outbreaks promptly and ensure the resilience of healthcare systems against future pandemics.

## 1. Introduction

The coronavirus disease 2019 (COVID-19) has emerged as one of the most significant global health challenges of our time. First identified in Wuhan, China, in December 2019, the virus quickly spread across international borders, leading to a pandemic declaration by the World Health Organization (WHO) in March 2020 [1]. The first confirmed case of COVID-19 in Europe was documented in France on 24 January [2], with Slovakia officially reporting its first confirmed case six weeks later on 6 March 2020 [3]. Furthermore, as of mid-May 2023, the WHO has reported a global total of 0.766 billion confirmed COVID-19 cases, with Europe accounting for 0.276 billion cases [4].

During the period of 2020–2022, Slovakia may have been affected by several COVID-19 strains, including variants of concern such as the B.1.1.7 and B.1.351 strains, which were first reported in the UK and South Africa, respectively [5]. Slovakia may have also been affected by other COVID-19 strains such as a variant named B.1.258 Δ (approx. 59% out of 251 sequenced samples between September and December 2020); this variant has spread and gained significant prevalence in multiple countries, including the Czech Republic, Sweden, Slovakia, Poland, Denmark, and Austria [6].

As countries around the world grappled with the unprecedented impact of the disease, it became crucial to study its spatial and temporal distribution to understand its patterns and guide effective mitigation strategies. Several studies have reported the spatiotemporal spread of COVID-19 in different countries. For example, a study employing a model to examine the spatiotemporal pattern of COVID-19 transmission in the northern provinces of Italy during the initial wave in Spring 2020 revealed a significant correlation between the spread of the virus and highway connections [7]. Another study used neighborhood matrices to model the impact of border restrictions between Switzerland and Italy on the spatiotemporal spread of the virus, finding that imposing restrictions decreased the number of cases by 12% compared to no border closure, while no restrictions increased the cases by a 2.7 factor [8]. Furthermore, the implementation of geographically weighted regression models demonstrated that the distribution pattern of COVID-19 exhibited strong spatial clustering. The study revealed that the highest-risk clusters of the disease were primarily concentrated in Central and Western Europe. In Indonesia, the transmission patterns of COVID-19 during first half of the outbreak (March to August of 2020) revealed that all provinces were affected by the virus during a short period of time [9]. Furthermore, after considering various variables, it was determined that people living in poverty and elderly populations were most substantially impacted by COVID-19, as they exhibited a significant relationship with the disease [10]. A recent study in Brazil [11] showed that clusters of confirmed cases were carried from a well-developed neighborhood to socially deprived areas, along with the emergence of hotspots of the fatality rate. The influence of age groups, income, level of education, and access to essential services on the spread of COVID-19 was also verified. The recognition of variables that influence the spatial spread of the disease are vital for pinpointing the most vulnerable areas. Therefore, understanding the spatial and temporal spread of the COVID-19 outbreak is critical to predicting local outbreaks and developing public health policies during the early stages of COVID-19. In Slovakia (a landlocked country in Central Europe), the spread of the virus has presented unique challenges. The available literature shows only one spatiotemporal investigation on COVID-19 in Slovakia [12]. The study revealed that the distribution of COVID-19 cases in Slovakia is characterized by spatial heterogeneity. By examining the distribution patterns at the district level, this study aims to gain insights into the localized dynamics of the disease within the country. Analyzing the spatial and temporal distribution of COVID-19 cases can provide valuable information on spread patterns, hotspots, clusters, and the effectiveness of control measures implemented in different districts. Therefore, this manuscript aims to provide a detailed examination of the spatial and temporal distribution of COVID-19 cases among five age groups (≤19 years) within different districts of Slovakia. By shedding light on the localized patterns and dynamics of the disease, the study contributes to ongoing efforts to control and manage the impact of the pandemic, both within the country and globally.

## 2. Materials and Methods

Data were obtained from the National Health Information Centre (NCZI), a state-funded organization founded by the Ministry of Health of the Slovak Republic for the period spanning from 12 March 2020 to 20 July 2022 covering 72 districts (8 regions) across Slovakia. Collected data of related COVID-19 infections encompassed the following cases: (1) Polymerase Chain Reaction (PCR) positive tests, positive antigen tests, and in the early stages, positive cases measured clinically by respiratory infection, (2) hospitalized patients, (3) admissions to intensive care units (ICUs), (4) patients who received pulmonary ventilation (PV), and (5) deaths. The records were specifically limited to five age groups: <1, 1–4, 5–9, 10–14, and 15–19 years. 

The coordinates (latitude and longitude) of the locations of PCR-positive, antigen test-positive, and hospitalized cases within different districts of the country were converted into shape files and geospatial maps of the total number of cases in different districts within the country were created using geographic information system (GIS) software (version 10.0). Each location is linked to its respective district boundary, and infection cases were assigned to their respective location within each district. The total number of cases in each district (on a three-month basis) were assigned different colors represent varying levels of infection rates. The total number of cases were separated into 6 categories: (1) no infections (0 cases), (2) <10, (3) 11–100, (4) 101–500, (5) 501–1000, and (6) >1000. 

The maps were then used to explore the significance of changes in tracking COVID-19 infections within different districts, focusing on their regular updates every three months (spatial and temporal). 

Furthermore, PCR positive cases, positive antigen tests, and in the early stages, positive cases measured clinically by respiratory infection, hospitalized cases, admissions to ICU, PV, and deaths were summarized for each district/region. Tables in Appendix A include a summary for total cases, cases in each age group, cases in each district and region, PCR positive cases, and hospitalizations per district area and population.

The criteria and measures for admission to hospitals, ICU, and PV are as follows: For the PCR test and antigen test, admission is based on suspected COVID-19 cases or positive antigen tests. Children who have been in contact with a suspected case within 2–14 days before the illness started, even if they are asymptomatic, are also admitted if they test positive or exhibit indicators such as fever, respiratory or digestive symptoms, and fatigue. Elevated markers of inflammation, liver enzyme abnormalities, and certain immunological responses are also considered. Imaging techniques like computed tomography (CT) scans, X-rays, and ultrasound examinations are used to detect lung damage. Hospitalization criteria include tachypnea without hypoxemia, oxygen saturation above 94% on atmospheric air, and chronic health problems or young age increasing the risk. Moderate pneumonia symptoms without hypoxemia or respiratory failure, as well as asymptomatic patients with positive CT scan findings, are also considered. Pediatric intensive care unit (PICU) admission criteria include oxygen saturation below 94%, severe febrile illness, dyspnea, tachypnea with cyanosis and oxygen hyposaturation (<92%), illness progression over 7–10 days, and evidence of organ dysfunction. Artificial PV is indicated for refractory hypoxia, respiratory insufficiency, and COVID-19 associated with acute respiratory distress syndrome (ARDS). High severity COVID-19 cases with complications such as shock, coagulation disorders, and organ failures also require ventilation. Severe diarrhea and illness progression over 7–10 days are additional factors for consideration.

## 3. Results

### 3.1. Data in General

**Figure 1:**
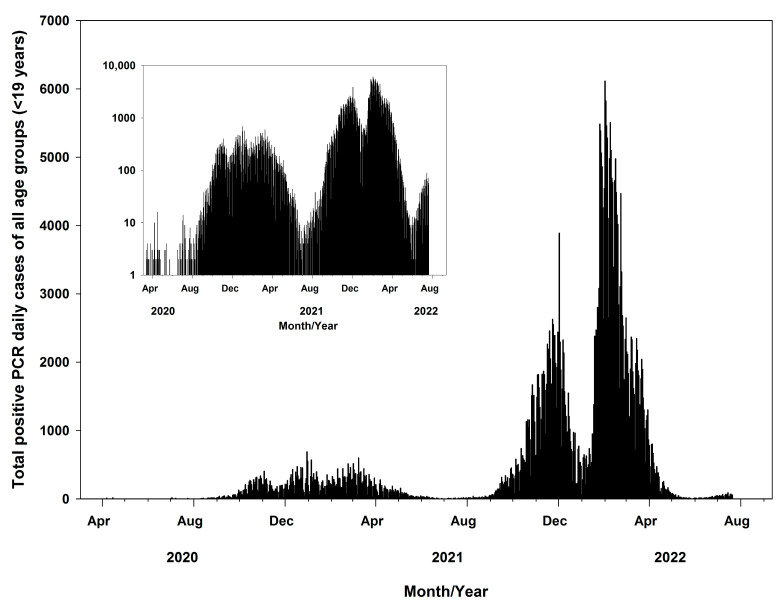
Evolution of daily PCR-confirmed COVID-19 cases in Slovakia during 12 March 2020 to 20 July 2022.

The total positive daily PCR cases all over the country were drawn; as shown in Figure 1, the inside figure shows total cases in a logarithmic scale. The increase in total cases shows an exponential trend with three peaks occurring during the study period. The first peak occurred between September 2021 and January 2022, the second between January 2022 and March 2022, and the third between March 2022 and April 2022. The maximum total number of cases were observed on 2 December 2021 (3889/393,429, 1%), the second on 1 February 2022 (6114), and the third on 1 March 2022 (2648/393,429, 0.7%). Furthermore, during the early stages of the pandemic (October 2020 to May 2021), the total daily cases were below 700 all over the country. 

A total of 393,429 positive PCR COVID-19 test cases or positive antigen test cases for all age groups were reported in Slovakia (Table A1, Appendix A). In addition, out of the total positive (393,429) cases, 18,886 (4.8%) were hospitalized, 456 (0.12%) individuals were admitted to the (PICU), 405 (0.10%) patients received pulmonary ventilation, and only 16 (0.004%) died ([Table A2, Table A3 and Table A4], Appendix A). The breakdown of the total positive cases by age group was as follows: <1-year-old group 2819 (0.72%), 1–4-year-old group 27,855 (7.08%), 5–9-year-old group 99,000 (25.16%), 10–14-year-old group 137,989 (35.07%), and 15–19-year-old group 125,766 (31.97).

The total positive PCR cases in 2020, 2021, and 2022 were 20,723, 14,6200, and 226,506, with the highest infections occurring in the Prešov 3703 (17.9%), 25,321 (17.3%), and Bratislava regions (37,444, 16.5%), respectively. 

On a monthly basis, the highest number of cases in 2020 were in December (8618) in Trenčín (1594, 18.5%), while in 2021 and 2022, the highest number of cases were in November (53,700) in Prešov (9027, 16.8%), and in February (107,829) in Žilina (15,696, 14.6%), respectively. 

Out of the total cases and for the whole period (393,429), the top three ranked regions were Prešov (62,377, 15.8%), Bratislava (58,635, 14.9%), and Žilina (58,361, 14.8%). On an age group basis, the highest number of infections were in the 10–14-year-old group (137,989, 35.1%) followed by the 15–19-year-old group (125,766, 32.0%), while on a yearly basis, 2020 had the highest number of infections (8122/20,723, 39.2%) for the 15–19-year-old group, and 2021 (53,351/146,200, 36.5%) and 2022 (78,029/226,506, 34.4%) had the highest number of infections for the 10–14-year-old group. In addition, for 2020 (according to the total cases of all age groups in each region), Prešov has the highest numbers (1575/3703, 42.5%) for the 15–19-year-old group; in 2021 the highest number of infections were in Prešov (9335/25,321, 36.9%) for the 10–14-year-old group, and in 2022, Bratislava ranked first (13,025/37,444, 34.9%) for the 10–14-year-old group. 

The highest number of hospitalized cases (for the whole period) were in the <1-year-old group (6405 out of 18,886, 33.9%). On an age group basis, the highest admissions were in the 15–19-year-old group in 2020 (575 out of 1321, 43.5%) and 2021(1940 out of 6629, 29.3%), respectively, while in 2022, a total of 4525 out of 10,936 (41.4%) of cases in the <1-year-old group were hospitalized. Furthermore, the highest admissions (region wise) in 2020 were in Bratislava (318 out of 1321, 24.1%), in 2021 (1955 out of 6629, 29.5%) in Košice, and in 2022 (1672 out of 10,936, 15.3%) in Nitra (Table A2, Appendix A). In addition, the PICU data (Table A3, Appendix A) show that the highest admissions were in the <1-year-old group (195 out of 456, 42.8%), with the majority occurring in Nitra (140, 71.8%), followed by the 15–19-year-old group (110, 24.1%), mostly in Košice (33, 30.0%). Pulmonary ventilation data (Table A4, Appendix A) show that the highest admissions (162/405, 40.0%) occurring in the <1-year-old group were mostly in Nitra (140/162, 86.4%), followed by the 10–14-year-old group (99/405, 24.4%), out of which almost half (46/99, 46.5%) occurred in Žilina. 

Cases by district and region per population and per area (case/km^2^) were determined for PCR antigen tests and hospitalized data (Table A1 and Table A2, Appendix A). On average, the whole country had 7.2% (393,429 case/5,459,781 population) and 8.3 (case/km^2^). In terms of regions (Table 1), Bratislava had the highest case/population (8.7%), while the lowest was in Nitra (5.5%). In terms of cases/km^2^, Bratislava came in first with 28.6, while Banská Bystrica came in last (3.9) (Table 1). 

In terms of districts, Senec was the highest with 11,540 cases/94,577 population (12.2%) while the lowest was Medzilaborce–Prešov (294 cases/11,708 pop., 2.5%). For case/km^2^ (district wise), the highest was Bratislava (34,624 case/365 km^2^, 94.8) and the lowest was Medzilaborce–Prešov (294 case/472 km^2^, 0.7) (Table A1, Appendix A).

**Figure 2:**
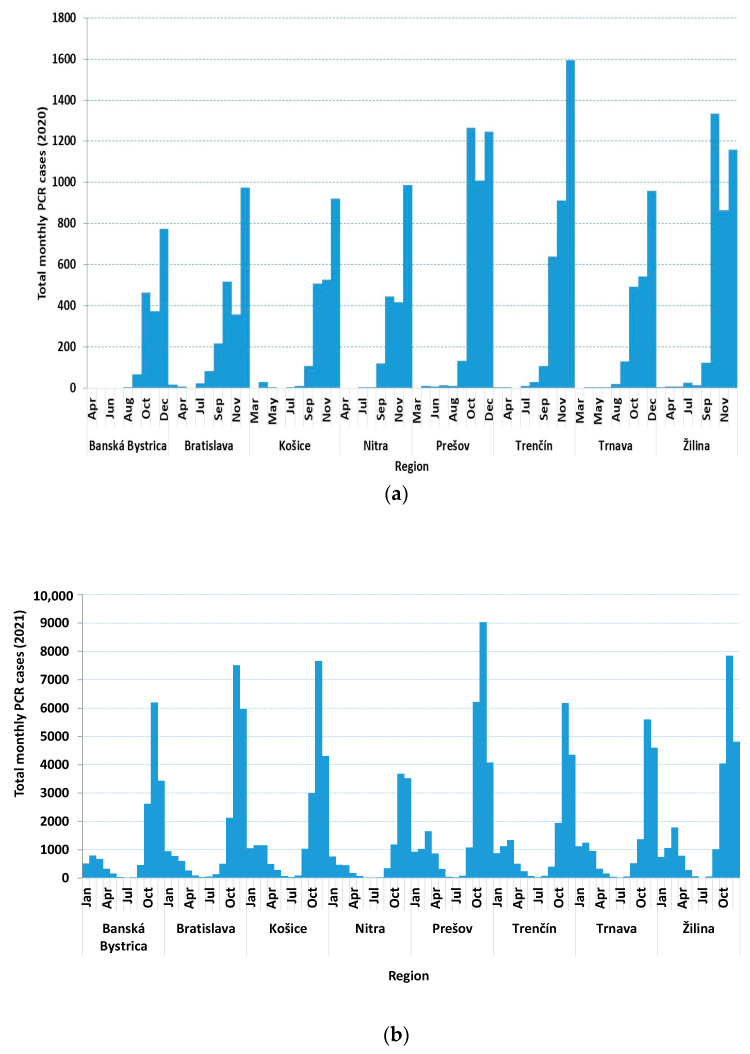

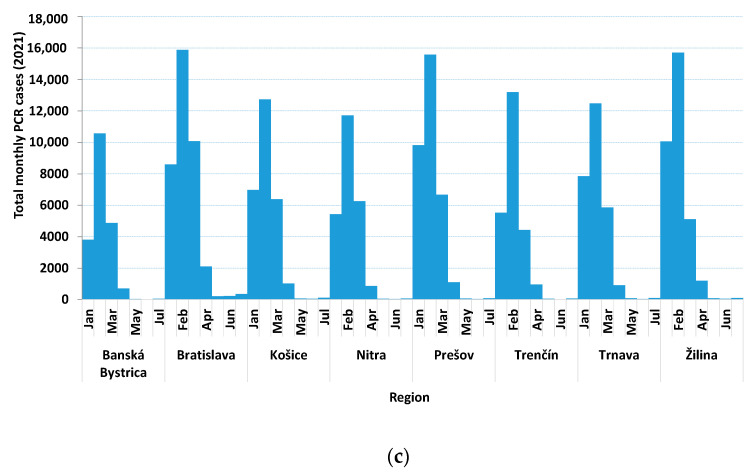
(**a**) Total positive monthly PCR cases in Slovakia during 2020. (**b**) Total positive monthly PCR cases in Slovakia during 2021. (**c**) Total positive monthly PCR cases in Slovakia during 2022.

The total PCR cases on a monthly basis for each region for 2020, 2021, and 2022 (Figure 2a–c) were also drawn. Figure 2a shows that the highest total monthly cases for 2020 were in Trenčín during December (1594); in addition, some regions show two peaks occurring during October and December in Prešov and Žilina, respectively. For 2021 (Figure 2b), the highest numbers were in Prešov (7849) and Žilina (9027) during November. Furthermore, during 2022 (Figure 2b), the highest numbers were observed during February in Bratislava (15,881), Žilina (15,696), and Prešov (15,576).

### 3.2. Spatiotemporal Mapping of COVID Cases

**Figure 3:**
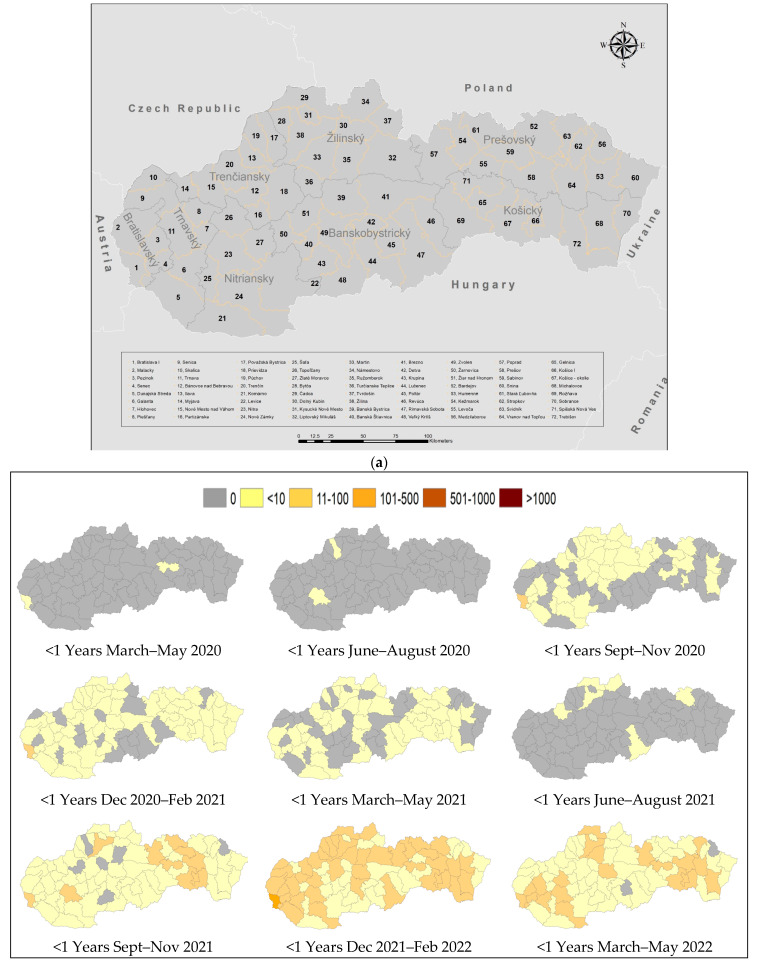

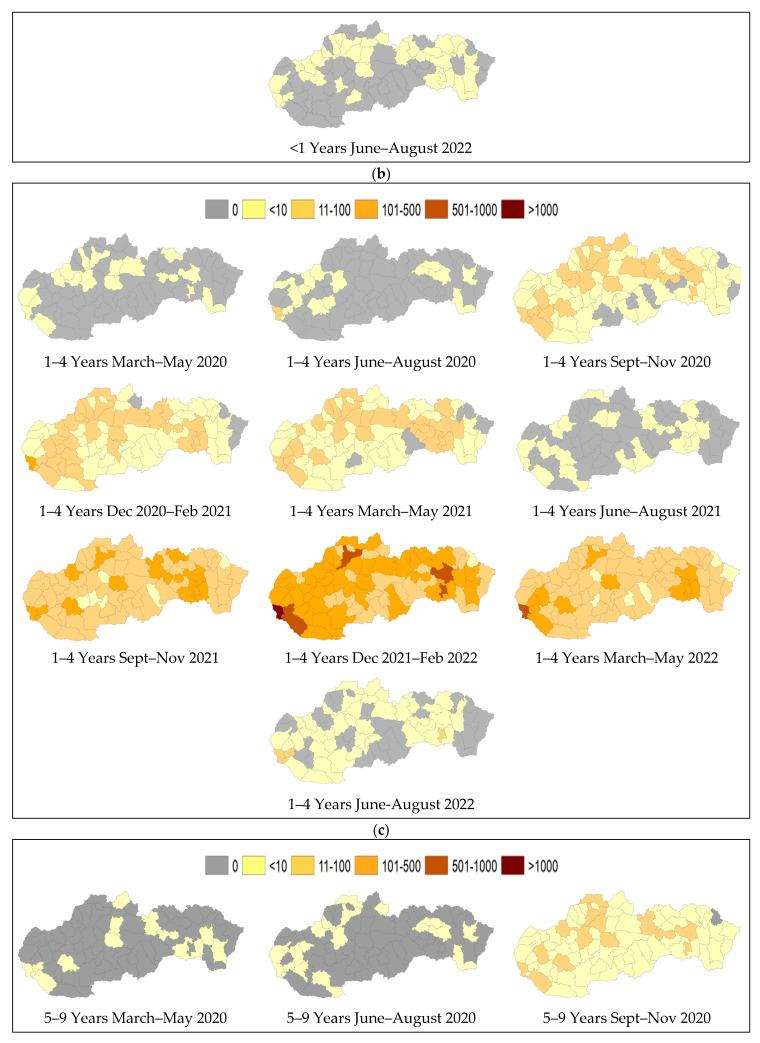

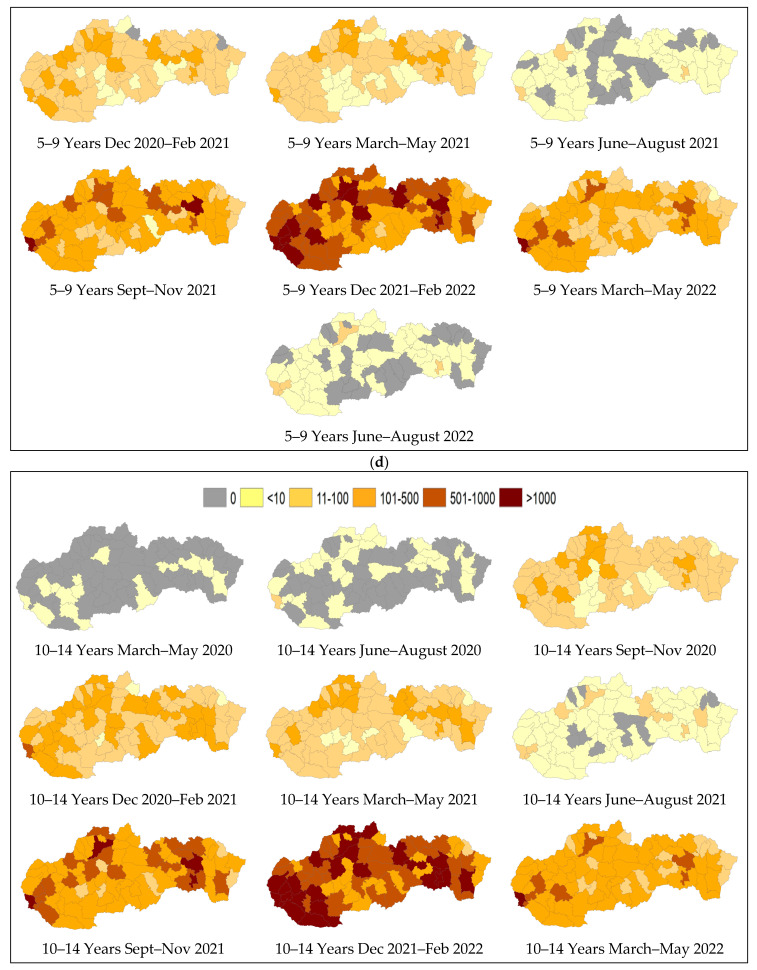

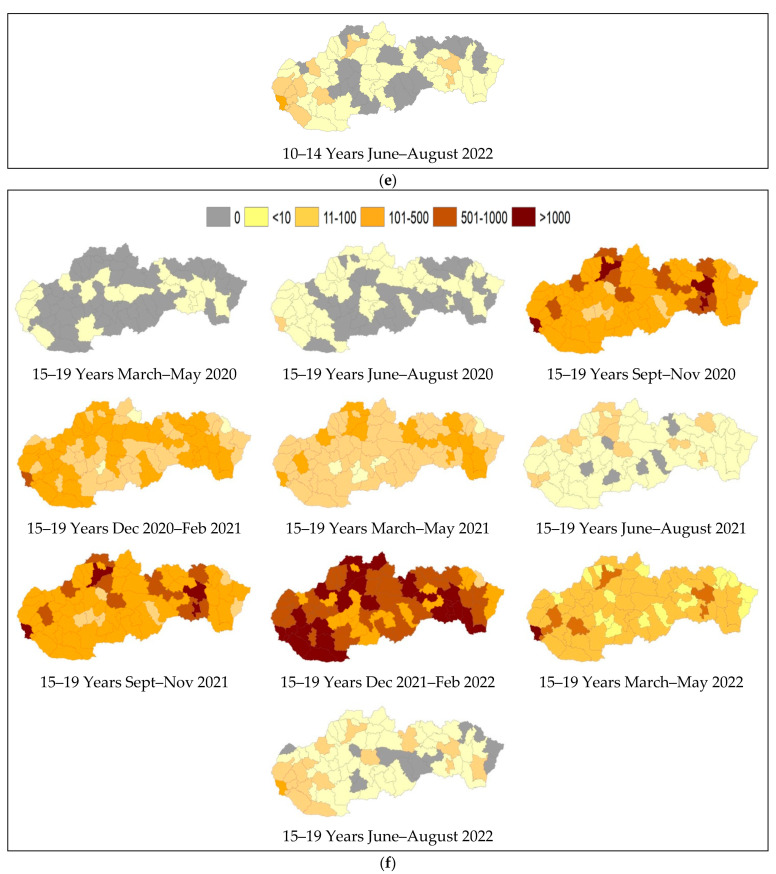
(**a**) Districts of Slovakia. (**b**) Spatiotemporal spread of positive PCR COVID-19 cases in the <1-year-old group based on three-month intervals in Slovakia. (**c**) Spatiotemporal spread of positive PCR COVID-19 cases in the 1–4-year-old group based on three-month intervals in Slovakia. (**d**) Spatiotemporal spread of positive PCR COVID-19 cases in the 5–9-year-old group based on three-month intervals in Slovakia. (**e**) Spatiotemporal spread of positive PCR COVID-19 cases in the 10–14-year-old group based on three-month intervals in Slovakia. (**f**) Spatiotemporal spread of positive PCR COVID-19 cases in the 15–19-year-old group based on three-month intervals in Slovakia.

Spatial maps for five age groups were created based on three-month intervals to provide a comprehensive visual representation of the spread of COVID-19 across the country’s districts over a span of two years (Figure 3a). Analyzing the spatial maps over these intervals reveals distinct patterns and trends in the spread of COVID-19. For example, during the early months of the pandemic, maps show few isolated clusters of cases in specific districts, mainly in Bratislava and Košice. These clusters are centered around major urban areas or regions with international travel connections, where the virus was likely introduced. As the pandemic progressed, the virus gradually spread to neighboring districts, both in urban and rural regions, reflecting the movement of infected individuals. Maps also show that the southern districts bordering Hungary have less infected cases (with time) compared to other regions, while regions bordering Czech Republic and Poland have higher cases than other regions. This trend is mainly discernable in the 10–14 and 14–19-year-old age groups. 

During the early stages of pandemic, maps of the <1-year-old group show only two districts (district–region) were affected during the first three months (Bratislava IV–Bratislava, Detva–Banská Bystrica) followed by (Bytča–Žilina and Nitra—Nitra). The virus then spread to most (about 70%) of the country’s districts (Sep 2020 to May 2021). Furthermore, during June to August 2021, the spread was only present in six districts, mainly in the northern parts of the country. During December 2021 to February 2022, maps show that the whole country was infected, where the eastern and northern parts of the country and major areas in the western part of the country show 11–100 cases, while the rest show <10 cases per three-month period, and only one district (Braislava IV) shows 101–500 cases (Figure 3b). 

For the 1–4-year-old group, similar trends were observed with more areas having higher numbers of infections. Figure 3c shows that the virus spread over the whole country during Sep 2021 to May 2022. During December 2021 to February 2022, maps for the 1–4-year-old group show four districts with 500–1000 cases (Senec, Žilina, Prešov, and Košice) and one with >1000 (Bratislava). 

Furthermore, the 5–9, 10–14, and 15–19-year-old age groups (Figure 3d–f) showed more areas with >1000 cases affected by the virus, especially during December 2021 to February 2022. For the 5–9, 10–14, and 15–19-year-old age groups, there were 14, 24, and 22 districts exceeding 1000 cases, and 28, 28, and 31 districts with 501–1000 cases, respectively.

## 4. Discussion

The selection of pediatric and teenage age groups was due to the comparatively milder impact of the pandemic on children, making it rare to observe fatalities. This choice aims to better grasp the spread of COVID-19 within these age groups/demographics.

The UN Convention on the Rights of the Child in the Slovak Republic defines a child as any individual under the age of 19 years, unless otherwise stipulated by the applicable law. Furthermore, a commonly used categorization for different stages of childhood and adolescence is infants (<1 year), young children (1–4 years), children (5–9 years), pre-adolescents (10–14 years), and adolescents (15–19 years). These age groupings are widely recognized and applied in various fields such as healthcare, education, and developmental psychology.

The spatiotemporal maps of Slovakia across the five age groups offer a revealing insight into the spread of COVID-19 over the study period. The maps, segmented into three-month intervals, demonstrate evolving patterns in the virus transmission. Regular updates every three months allow for the monitoring of infection patterns and trends in different districts; in addition, spatial maps can identify districts with higher or lower cases and the presence of hot spots at specific times. 

Initially, during the early stages of the pandemic, isolated clusters of cases were primarily observed in major urban hubs like Bratislava and Košice, likely due to international travel connections [13]. As time progressed, the virus spread to neighboring urban and rural districts, indicating the movement of infected individuals. Notably, the southern districts bordering Hungary showed fewer infections over time compared to regions bordering Czech Republic and Poland; this was particularly evident in the 10–14 and 14–19-year-old age groups. In the <1-year-old group, the initial impacts were limited to a few districts and gradually encompassed approximately 70% of the country’s districts by May 2021. However, a resurgence in infections was evident from June to August 2021, mainly in the northern part of the country. By December 2021 to February 2022, the entire country was affected, with varying intensity across regions. The 1–4-year-old age group exhibited similar trends but with broader geographical coverage and higher infection numbers, including districts with 500–1000 cases and one district with over 1000 cases. Moreover, the 5–9, 10–14, and 15–19-year-old age groups showed widespread infected areas, notably peaking during December 2021 to February 2022, with several districts surpassing 1000 cases and a significant number of districts reporting between 501 and 1000 cases. These findings underscore the varied temporal and spatial dynamics of COVID-19 transmission across different age groups in Slovakia, highlighting the need for targeted intervention strategies and public health measures to mitigate the impact of the pandemic.

The spread of COVID-19 in Slovakia, as depicted in the spatiotemporal maps across different age groups, can be attributed to several factors. Initially, the emergence of isolated clusters in major urban centers such as Bratislava and Košice suggests that the virus was likely introduced through international travel connections, a common pathway for global early infections [13,14]. As the pandemic progressed, factors such as population density, mobility within and between regions, and social interactions likely facilitated the virus’s spread to urban and rural neighboring districts [15]. The observed patterns of infection in districts bordering Hungary, Czech Republic, and Poland could be influenced by cross-border travel and interactions, highlighting the importance of regional dynamics in the virus transmission [16]. Additionally, varying levels of adherence to preventive measures, such as closing preschools, primary and secondary schools, mask-wearing, social distancing, and testing availability, could have impacted infection rates across different areas and age groups. The resurgence of infections in specific regions during certain periods, such as the northern region in mid-2021, may reflect localized outbreaks linked to events or gatherings. Moreover, age-specific behaviors and activities could contribute to the observed differences, with teenage groups possibly engaging in more social interactions and activities, leading to higher infection rates. Overall, the complex interplay of geographic, demographic, behavioral, and preventive factors shapes the spatial and temporal dynamics of COVID-19 transmission in Slovakia, emphasizing the need for tailored interventions and vigilant public health measures to curb the spread of the virus.

According to the available data and research, there are several factors that contribute to the higher COVID-19 infection rates among individuals under the age of 19. (1) Behavioral patterns: teenage individuals generally tend to engage in more social interactions than younger ones, which increases their chances of coming into contact with the virus. This age group may be more likely to disregard preventive measures (e.g., social distancing and mask-wearing) [17,18,19]. (2) School settings: educational institutions, such as schools and colleges, provide an environment where the virus can easily spread among students due to close contact and shared spaces [20]. (3) Asymptomatic cases: younger individuals are more likely to be asymptomatic, meaning they do not exhibit typical COVID-19 symptoms even if they are infected. This makes it harder to identify and isolate cases, leading to further spread within their communities [21]. 

Therefore, these maps offer valuable insights into the temporal and spatial dynamics of the pandemic, allowing policymakers, healthcare professionals, and the general public to track the progression of the virus and make data-driven decisions. Hotspots, depicted as areas with high infection rates, shift across districts as localized outbreaks occur and are brought under control through targeted measures. These maps also reveal the impact of various factors, such as population density, socioeconomic conditions (urban vs. rural areas), and healthcare infrastructure, on the spread and containment of the virus within different districts [22,23].

By examining the maps over the study period, it becomes evident that the spread of COVID-19 is not uniform throughout the country. Some districts had multiple waves of infections, while others managed to maintain a relatively lower number of cases. This highlights the importance of localized strategies and interventions tailored to specific districts, considering their unique characteristics and vulnerabilities. A detailed spatiotemporal analysis in six periods based on six-month intervals by [12] using the spatial autocorrelation of COVID-19 in Slovakia showed that the spatial distribution of COVID-19 in Slovakia is heterogeneous, and cases were concentrated in all regions depending on the period in which they were monitored. In addition, their research suggested that geographic and temporal analyses will be very important in the future. 

A study conducted on eight age groups (<20, 20–29, 30–39, …, and >80 years) revealed that neighborhood socioeconomic status was a more significant risk factor for COVID-19 incidence in children and working-age adults compared to seniors [24]. Social demographics and housing conditions were important risk factors for COVID-19 incidence in older age groups. Additionally, transportation-related variables showed significant associations with COVID-19 incidences across multiple age groups. The study concluded that age played a role in modifying the relationship between neighborhood characteristics and COVID-19 incidence. 

A geographic information system (GIS) modeling approach was employed to pinpoint the risk factors influencing COVID-19 incidence rates at the district level. The model outcomes indicated that urban population and proximity to the capital city were significant factors contributing to the COVID-19 incidence rates in Bangladesh. Moreover, the study revealed that urban areas exhibited a higher prevalence of COVID-19 infections compared to rural areas [25]. 

Demographic studies provide a good understanding of the spread and fatality rates of COVID-19. Several demographic factors contribute to the age-specific infection rates. The main factor is likely to be age itself. Older individuals tend to have a higher risk of contracting the virus due to their weakened immune system and preexisting health conditions (e.g., heart disease, diabetes, or respiratory issues). Other demographic factors, such as healthcare workers or professions that require close contact with others, may increase the risk of exposure to the virus. In addition, living conditions and socioeconomic factors can also impact the likelihood of infection, as individuals in crowded households or low-income communities may have limited access to preventive measures like social distancing or healthcare [26]. 

A study explored the spatial association between socio-demographic factors and COVID-19 outcomes across Europe [27]. In Slovakia, this study showed moderate associations between socio-demographic variables (such as income and poverty) and COVID-19 cases and deaths. This suggests that factors like total population, poverty, and income play a significant role in regulating COVID-19 casualties. The analysis also indicated heterogeneous distributions of cases and deaths across Europe, with Western European countries showing higher actual values. The study underlined the importance of considering infection rates and recovery rates for a more accurate interpretation of COVID-19 impacts. Additionally, spatial regression models highlighted a strong positive association between income, total population, and COVID-19 cases and deaths in European countries, suggesting these as key controlling variables for pandemic outcomes.

In the context of Slovakia, exploring the spatial association between socio-demographic factors and COVID-19 outcomes can provide valuable insights into the potential drivers of transmission and the severity of the disease. By analyzing the geographic distribution of COVID-19 cases, we can identify areas with higher incidence rates and investigate the socio-demographic characteristics that may be associated with these hotspots. This information can help policymakers and health authorities tailor their response efforts to target vulnerable populations and mitigate the further spread of the virus. 

Furthermore, the policy of admitting children and adolescents to the hospital in general is easy to uphold, especially during the pandemic in Slovakia; even so, all the admitted cases had symptoms of respiratory infection or gastrointestinal acute disorders like diarrhea and vomiting. Otherwise, the referral of severe COVID-19 cases from small, rural hospitals to large children’s hospitals was common; that is why the number of intensive care unit admissions and patients receiving pulmonary ventilation in large teaching children’s hospitals significantly increased in comparison to those in smaller hospitals (See Table A3, Appendix A), like hospitals in Nitra, Košice, and Bratislava. 

The spatial maps offer a crucial retrospective tool for assessing intervention effectiveness in districts during the COVID-19 pandemic. Policymakers can correlate intervention timing with map patterns to identify successful containment measures and areas needing further attention [23]. However, these maps rely on evolving data sources, which are potentially affected by testing capacity and reporting discrepancies among districts, impacting data reliability. This underscores the need for comprehensive data for decision-making. Disparities in testing access can lead to varying case reports, affecting our grasp of the virus’s spread. To enhance understanding, these maps should be used alongside other data and local knowledge. Overall, these maps aid in monitoring, analyzing, and responding to COVID-19, and guiding targeted interventions and resource allocation to safeguard public health [28,29].

This study presented a comprehensive analysis of the spatiotemporal patterns of COVID-19 infections across five ≤19-year-old age groups in 72 districts of Slovakia from March 2020 to July 2022. The findings highlight the significant impact of the pandemic on the country and provide valuable insights for understanding the disease’s dynamics.

One limitation of this study is the potential concern arising from the substantial loss of outpatient data, especially for patients recruited from hospital settings, which could result in selection bias and affect the validity of the study’s findings; however, in this case, the available data were only analyzed for registered cases.

## 5. Conclusions

Spatial mapping showed high-risk infection clusters mainly in urban areas with gradual spread to neighboring districts. The highest infections were associated with pre-adolescent and adolescent groups. The study highlighted the impact of population density and socioeconomic conditions on the spread and containment of the virus. These insights are vital for targeted public health strategies and resource allocation.

## Figures and Tables

**Table 1:** Case per population and case per area (km^2^) of all regions of Slovakia.

Region	Case/Pop %	Case/km^2^
Banská Bystrica	5.8	3.91
Bratislava	8.7	28.63
Košice	6.2	7.38
Nitra	5.5	5.85
Prešov	7.5	6.96
Trenčín	7.7	9.93
Trnava	8	10.97
Žilina	8.4	8.58

## Data Availability

Original and generated data for study are available from A.G. on reasonable request.

## Appendix A

**Table A1:** Total PCR cases in different age groups, case per population, and case per area (km^2^) of all regions and districts of Slovakia.

	Age Group (Year)									
Region/District	<1	1–4	5–9	10–14	15–19	Total	Pop	Area km^2^	Case/Pop %	Case/km^2^
**Banská Bystrica**	**226**	**2377**	**8539**	**13,037**	**12,806**	**36,985**	**643,102**	**9450**	**5.8**	**3.91**
Banská Bystrica	47	672	2287	3252	2837	9095	110,631	809	8.2	11.24
Banská Štiavnica	8	69	210	336	300	923	16,003	292	5.8	3.16
Brezno	8	180	679	1114	1123	3104	60,905	1265	5.1	2.45
Detva	15	151	548	715	776	2205	31,771	449	6.9	4.91
Krupina	9	96	279	434	505	1323	22,050	584	6.0	2.27
Lučenec	16	149	757	1289	1220	3431	73,071	825	4.7	4.16
Poltár	3	46	187	342	377	955	21,179	476	4.5	2.01
Revúca	8	64	255	475	588	1390	39,349	730	3.5	1.90
Rimavská Sobota	24	208	814	1351	1365	3762	83,953	1471	4.5	2.56
Veľký Krtíš	15	91	408	681	776	1971	43,002	848	4.6	2.32
Žarnovica	14	81	266	392	375	1128	26,054	425	4.3	2.65
Žiar nad Hronom	18	196	664	923	942	2743	46,477	517	5.9	5.31
Zvolen	41	374	1185	1733	1622	4955	68,657	759	7.2	6.53
**Bratislava**	**422**	**5150**	**17,314**	**20,716**	**15,033**	**58,635**	**677,024**	**2048**	**8.7**	**28.63**
Bratislava	276	3132	10,338	12,084	8794	34,624	440,948	365	7.9	94.86
Malacky	30	312	1429	2211	1713	5695	75,325	949	7.6	6.00
Pezinok	36	545	1937	2452	1806	6776	66,174	375	10.2	18.07
Senec	80	1161	3610	3969	2720	11,540	94,577	359	12.2	32.14
**Košice**	**402**	**3532**	**11,770**	**17,543**	**16,580**	**49,827**	**802,092**	**6749**	**6.2**	**7.38**
Gelnica	17	92	360	485	479	1433	31,923	584	4.5	2.45
Košice	145	1482	4423	6034	5407	17,491	238,138	241	7.3	72.58
Košice—okolie	69	634	1894	2878	2681	8156	131,305	1534	6.2	5.32
Michalovce	50	360	1376	2119	2043	5948	110,670	1019	5.4	5.84
Rožňava	14	145	653	1140	1066	3018	61,944	1173	4.9	2.57
Sobrance	7	63	188	336	394	988	22,775	538	4.3	1.84
Spišská Nová Ves	62	541	1930	2532	2216	7281	100,201	587	7.3	12.40
Trebišov	38	215	946	2019	2294	5512	105,136	1073	5.2	5.14
**Nitra**	**260**	**2348**	**8707**	**13,262**	**12,549**	**37,126**	**671,508**	**6341**	**5.5**	**5.85**
Komárno	23	234	1121	1906	1697	4981	100,992	1100	4.9	4.53
Levice	24	215	904	1541	1530	4214	110,040	1551	3.8	2.72
Nitra	102	925	3140	4096	3777	12,040	161,499	870	7.5	13.84
Nové Zámky	52	360	1337	2121	2097	5967	137,778	1347	4.3	4.43
Šaľa	14	245	817	1170	1050	3296	51,309	355	6.4	9.28
Topoľčany	35	268	997	1768	1749	4817	69,521	597	6.9	8.07
Zlaté Moravce	10	101	391	660	649	1811	40,369	521	4.5	3.48
**Prešov**	**511**	**4330**	**15,160**	**21,693**	**20,683**	**62,377**	**827,028**	**8965**	**7.5**	**6.96**
Bardejov	38	314	1246	2016	2198	5812	77,666	935	7.5	6.22
Humenné	45	220	763	1245	1268	3541	61,398	754	5.8	4.70
Kežmarok	28	265	1021	1588	1431	4333	76,165	629	5.7	6.89
Levoča	14	200	580	856	854	2504	33,730	421	7.4	5.95
Medzilaborce	3	17	47	87	140	294	11,708	427	2.5	0.69
Poprad	56	588	2237	2940	2567	8388	105,015	1104	8.0	7.60
Prešov	132	1323	4281	5300	4838	15,874	176,781	933	9.0	17.01
Sabinov	43	401	1328	1740	1542	5054	61,072	545	8.3	9.27
Snina	14	89	356	659	741	1859	35,833	804	5.2	2.31
Stará Ľubovňa	39	362	1317	1888	1694	5300	54,054	707	9.8	7.50
Stropkov	19	103	407	609	556	1694	20,366	388	8.3	4.37
Svidník	22	130	548	917	875	2492	32,334	549	7.7	4.54
Vranov nad Topľou	58	318	1029	1848	1979	5232	80,906	769	6.5	6.80
**Trenčín**	**252**	**2969**	**11,250**	**15,735**	**14,442**	**44,648**	**582,567**	**4497**	**7.7**	**9.93**
Bánovce nad Bebravou	7	204	847	1105	983	3146	35,972	461	8.7	6.82
Ilava	27	233	1064	1556	1380	4260	58,811	358	7.2	11.90
Myjava	11	181	624	781	652	2249	26,062	327	8.6	6.88
Nové Mesto nad Váhom	36	370	1352	1852	1585	5195	62,572	579	8.3	8.97
Partizánske	14	186	630	946	928	2704	45,293	301	6.0	8.98
Považská Bystrica	32	432	1363	1874	1787	5488	61,993	463	8.9	11.85
Prievidza	45	389	1453	2589	2603	7079	132,891	959	5.3	7.38
Púchov	34	239	949	1268	1284	3774	44,136	375	8.6	10.06
Trenčín	46	735	2968	3764	3240	10,753	114,837	674	9.4	15.95
**Trnava**	**282**	**3207**	**12,200**	**15,927**	**13,854**	**45,470**	**565,324**	**4144**	**8.0**	**10.97**
Dunajská Streda	49	818	2818	3536	2642	9863	123,355	1074	8.0	9.18
Galanta	41	385	1712	2271	2114	6523	94,179	641	6.9	10.18
Hlohovec	24	242	984	1290	1199	3739	44,731	267	8.4	14.00
Piešťany	47	448	1381	1783	1630	5289	62,626	381	8.4	13.88
Senica	30	324	1114	1628	1516	4612	60,314	683	7.6	6.75
Skalica	20	238	1061	1447	1301	4067	46,965	357	8.7	11.39
Trnava	71	752	3130	3972	3452	11,377	133,154	741	8.5	15.35
**Žilina**	**464**	**3942**	**14,060**	**20,076**	**19,819**	**58,361**	**691,136**	**6802**	**8.4**	**8.58**
Bytča	28	141	556	861	898	2484	31,056	281	8.0	8.84
Čadca	45	361	1460	2298	2716	6880	89,494	760	7.7	9.05
Dolný Kubín	33	172	683	1016	995	2899	39,480	491	7.3	5.90
Kysucké Nové Mesto	18	105	475	855	865	2318	32,890	173	7.0	13.40
Liptovský Mikuláš	21	389	1244	1824	1756	5234	72,054	1341	7.3	3.90
Martin	55	611	2326	2946	2708	8646	95,921	735	9.0	11.76
Námestovo	50	291	1171	1954	1950	5416	63,268	690	8.6	7.85
Ružomberok	32	298	1022	1487	1489	4328	56,536	646	7.7	6.70
Turčianske Teplice	4	68	286	367	361	1086	15,854	392	6.9	2.77
Tvrdošín	21	142	514	875	1003	2555	36,127	478	7.1	5.35
Žilina	157	1364	4323	5593	5078	16,515	158,456	815	10.4	20.26
**Grand Total**	**2819**	**27,855**	**99,000**	**137,989**	**125,766**	**393,429**	**5,459,781**	**48,996**	**7.2**	**8.3**

Values in bold indicate total cases in region.

**Table A2:** Total hospitalization cases in different age groups, case per population, and case per area (km^2^) of all regions and districts of Slovakia.

	Age Group (Year)									
Region/District	<1	1–4	5–9	10–14	15–19	Total	pop	Area km^2^	Case/Pop %	Case/km^2^
**Banská Bystrica**	**254**	**229**	**146**	**212**	**452**	**1293**	**643,102**	**9450**	**0.20**	**0.14**
Banská Bystrica	206	193	128	181	170	878	110,631	809	0.79	1.09
Brezno	15	2		4	19	40	60,905	1265	0.07	0.03
Lučenec					36	36	73,071	825	0.05	0.04
Revúca			8	8	5	21	39,349	730	0.05	0.03
Rimavská Sobota					5	5	83,953	1471	0.01	0.00
Veľký Krtíš	1		3	4	1	9	43,002	848	0.02	0.01
Žiar nad Hronom		3	2	8	204	217	46,477	517	0.47	0.42
Zvolen	32	31	5	7	12	87	68,657	759	0.13	0.11
Bratislava	746	1041	465	441	616	3309	677,024	2048	0.49	1.62
Bratislava	746	1041	465	441	585	3278	440,948	365	0.74	8.98
Pezinok					31	31	66,174	375	0.05	0.08
Košice	1227	1011	370	514	938	4060	802,092	6749	0.51	0.60
Košice	879	785	306	410	662	3042	31,923	584	9.53	5.21
Michalovce	5	13		40	103	161	110,670	1019	0.15	0.16
Rožňava	31	6	7	5	25	74	61,944	1173	0.12	0.06
Spišská Nová Ves	224	136	31	26	93	510	100,201	587	0.51	0.87
Trebišov	88	71	26	33	55	273	105,136	1073	0.26	0.25
Nitra	2443	242	103	74	219	3081	671,508	6341	0.46	0.49
Komárno	102	43	21	15	41	222	100,992	1100	0.22	0.20
Levice	67	131	17	6	51	272	110,040	1551	0.25	0.18
Nitra	2210	19	42	26	63	2360	161,499	870	1.46	2.71
Nové Zámky					8	8	137,778	1347	0.01	0.01
Topoľčany	64	49	23	27	56	219	69,521	597	0.32	0.37
Prešov	1175	776	305	696	1170	4122	827,028	8965	0.50	0.46
Bardejov	47	72	11	28	93	251	77,666	935	0.32	0.27
Humenné	130	34	15	3	32	214	61,398	754	0.35	0.28
Kežmarok	37	26		18	44	125	76,165	629	0.16	0.20
Levoča					3	3	33,730	421	0.01	0.01
Poprad	172	207	130	145	185	839	105,015	1104	0.80	0.76
Prešov	482	251	140	457	717	2047	176,781	933	1.16	2.19
Snina	35	16	3	23	26	103	61,072	545	0.17	0.19
Stará Ľubovňa	252	116	5	12	58	443	35,833	804	1.24	0.55
Svidník	20	54	1	10	12	97	32,334	549	0.30	0.18
Trenčín	171	156	41	70	416	854	582,567	4497	0.15	0.19
Myjava	6	8	3	4	28	49	26,062	327	0.19	0.15
Partizánske					4	4	45,293	301	0.01	0.01
Považská Bystrica	50	30			59	139	61,993	463	0.22	0.30
Prievidza	45	53	7	8	104	217	132,891	959	0.16	0.23
Trenčín	70	65	31	58	221	445	114,837	674	0.39	0.66
Trnava	154	171	57	67	126	575	565,324	4144	0.10	0.14
Dunajská Streda		7		3	13	23	123,355	1074	0.02	0.02
Galanta		1	1	11	18	31	94,179	641	0.03	0.05
Piešťany	48	38	19	39	31	175	62,626	381	0.28	0.46
Skalica		11			6	17	46,965	357	0.04	0.05
Trnava	106	114	37	14	58	329	133,154	741	0.25	0.44
Žilina	235	575	99	219	464	1592	691,136	6802	0.23	0.23
Čadca	59	44	20	9	65	197	89,494	760	0.22	0.26
Dolný Kubín	65	79	6	33	26	209	39,480	491	0.53	0.43
Liptovský Mikuláš	70	90	5	15	32	212	72,054	1341	0.29	0.16
Martin	23	238	38	148	250	697	95,921	735	0.73	0.95
Ružomberok		3		5	28	36	56,536	646	0.06	0.06
Tvrdošín	18	121	30	9	37	215	36,127	478	0.60	0.45
Žilina					26	26	158,456	815	0.02	0.03
**Grand Total**	**6405**	**4201**	**1586**	**2293**	**4401**	**18,886**	**5,459,781**	**48,996**	**0.35**	**0.39**

Values in bold indicate total cases in region.

**Table A3:** Total intensive care unit admissions in different age groups in all regions and districts of Slovakia.

	Age Group (Year)					
Region/District	<1	1–4	5–9	10–14	15–19	Total
**Banská Bystrica**	**9**	**0**	**13**	**6**	**4**	**32**
Banská Bystrica	9		13	6		28
Lučenec					4	4
**Bratislava**	**23**	**0**	**39**	**24**	**16**	**102**
Bratislava	23	0	39	24	16	102
**Košice**	**23**	**20**	**19**	**27**	**33**	**122**
Košice	14	19	7	27	18	85
Michalovce					8	8
Spišská Nová Ves	9	1	12			22
Trebišov					7	7
Nitra	140	0	0	2	16	158
Komárno					10	10
Levice				2	4	6
**Nitra**	**140**					**140**
Topoľčany					2	2
**Prešov**	**0**	**0**	**0**	**1**	**9**	**10**
Bardejov					4	4
Humenné				1		1
Stará Ľubovňa					5	5
**Trenčín**					**1**	**1**
Myjava					1	1
**Žilina**	**0**	**0**	**0**	**0**	**31**	**31**
Martin					20	20
Tvrdošín					11	11
**Grand Total**	**195**	**20**	**71**	**60**	**110**	**456**

Values in bold indicate total cases in region.

**Table A4:** Total pulmonary ventilation admissions in different age groups in all regions and districts of Slovakia.

	Age Group (Year)					
Region/District	<1	1–4	5–9	10–14	15–19	Total
**Banská Bystrica**		**2**			**1**	**3**
Banská Bystrica		2				2
Revúca					1	1
**Bratislava**	**1**		**38**	**10**	**6**	**55**
Bratislava	1		38	10	6	55
**Košice**	**12**	**19**	**1**	**43**	**25**	**100**
Košice	11	19		43	18	91
Spišská Nová Ves	1		1			2
Trebišov					7	7
**Nitra**	**140**				**10**	**150**
Komárno					6	6
Levice					4	4
Nitra	140					140
**Prešov**	**9**				**8**	**17**
Bardejov					1	1
Humenné	2					2
Poprad	7					7
Snina					7	7
**Trenčín**					**12**	**12**
Myjava					1	1
Považská Bystrica					3	3
Trenčín					8	8
**Žilina**				**46**	**22**	**68**
Čadca					3	3
Martin				46	17	63
Tvrdošín					1	1
Žilina					1	1
Grand Total	**162**	**21**	**39**	**99**	**84**	**405**

Values in bold indicate total cases in region.

## References

[1] Kang D., Choi H., Kim J.-H., Choi J. (2020). Spatial epidemic dynamics of the COVID-19 outbreak in China. Int. J. Infect. Dis..

[2] Stoecklin S.B., Rolland P., Silue Y., Mailles A., Campese C., Simondon A., Mechain M., Meurice L., Nguyen M., Bassi C. (2020). First cases of coronavirus disease 2019 (COVID-19) in France: Surveillance, investigations and control measures, January 2020. Eurosurveillance.

[3] IHME-Institute for Health Metrics and Evaluation (2022). COVID-19 Resources. http://www.healthdata.org/covid.

[4] WHO (2023). WHO Coronavirus (COVID-19) Dashboard.

[5] González-Candelas F., Shaw M.-A., Phan T., Kulkarni-Kale U., Paraskevis D., Luciani F., Kimura H., Sironi M. (2021). One year into the pandemic: Short-term evolution of SARS-CoV-2 and emergence of new lineages. Infect. Genet. Evol..

[6] B.1.258∆, a SARS-CoV-2 Variant with ∆H69/∆V70 in the Spike Protein Circulating in the Czech Republic and Slovakia-SARS-CoV-2 coronavirus/nCoV-2019 Genomic Epidemiology-Virological. (n.d.). https://virological.org/t/b-1-258-a-sars-cov-2-variant-with-h69-v70-in-the-spike-protein-circulating-in-the-czech-republic-and-slovakia/613.

[7] De Angelis M., Durastanti C., Giovannoni M., Moretti L. (2022). Spatio-temporal distribution pattern of COVID-19 in the Northern Italy during the first-wave scenario: The role of the highway network. Transp. Res. Interdiscip. Perspect..

[8] Grimée M., Dunbar M.B.-N., Hofmann F., Held L. (2022). Modelling the effect of a border closure between Switzerland and Italy on the spatiotemporal spread of COVID-19 in Switzerland. Spat. Stat..

[9] Aisyah D.N., Mayadewi C.A., Diva H., Kozlakidis Z., Siswanto, Adisasmito W. (2020). A spatial-temporal description of the SARS-CoV-2 infections in Indonesia during the first six months of outbreak. PLoS ONE.

[10] Kianfar N., Mesgari M.S. (2022). GIS-based spatio-temporal analysis and modeling of COVID-19 incidence rates in Europe. Spat. Spatio-Temporal Epidemiology.

[11] de Souza A.P.G., Mota C.M.d.M., Rosa A.G.F., de Figueiredo C.J.J., Candeias A.L.B. (2022). A spatial-temporal analysis at the early stages of the COVID-19 pandemic and its determinants: The case of Recife neighborhoods, Brazil. PLoS ONE.

[12] Vilinová K., Petrikovičová L. (2023). Spatial Autocorrelation of COVID-19 in Slovakia. Trop. Med. Infect. Dis..

[13] Jamal S., Paez A. (2024). Socio-economic and demographic differences in the impact of COVID-19 on personal travel in the Global South. Transp. Rev..

[14] Zoungrana T.D., Yerbanga A., Ouoba Y. (2022). Socio-economic and environmental factors in the global spread of COVID-19 outbreak. Res. Econ..

[15] Kwok C.Y.T., Wong M.S., Chan K.L., Kwan M.-P., Nichol J.E., Liu C.H., Wong J.Y.H., Wai A.K.C., Chan L.W.C., Xu Y. (2021). Spatial analysis of the impact of urban geometry and socio-demographic characteristics on COVID-19, a study in Hong Kong. Sci. Total. Environ..

[16] Vega-Gonzalo M., Gomez J., Christidis P. (2023). How has COVID-19 changed private car use in European urban areas? An analysis of the effect of socio-economic characteristics and mobility habits. Transp. Res. Part A Policy Pract..

[17] Irawan M.Z., Rizki M., Joewono T.B., Belgiawan P.F. (2020). Exploring the intention of out-of-home activities participation during new normal conditions in Indonesian cities. Transp. Res. Interdiscip. Perspect..

[18] Kim J.K., Crimmins E.M. (2020). How does age affect personal and social reactions to COVID-19: Results from the national Understanding America Study. PLoS ONE.

[19] Chen T., Lucock M. (2022). The mental health of university students during the COVID-19 pandemic: An online survey in the UK. PLoS ONE.

[20] Magson N.R., Freeman J.Y.A., Rapee R.M., Richardson C.E., Oar E.L., Fardouly J. (2021). Risk and Protective Factors for Prospective Changes in Adolescent Mental Health during the COVID-19 Pandemic. J. Youth Adolesc..

[21] Snape M.D., Viner R.M. (2020). COVID-19 in children and young people. Science.

[22] Andrews M.R., Tamura K., Best J.N., Ceasar J.N., Batey K.G., Kearse T.A., Allen L.V., Baumer Y., Collins B.S., Mitchell V.M. (2021). Spatial Clustering of County-Level COVID-19 Rates in the U.S. Int. J. Environ. Res. Public Health.

[23] Ficetola G.F., Rubolini D. (2021). Containment measures limit environmental effects on COVID-19 early outbreak dynamics. Sci. Total. Environ..

[24] Zhang X., Smith N., Spear E., Stroustrup A. (2021). Neighborhood characteristics associated with COVID-19 burden-the modifying effect of age. J. Expo. Sci. Environ. Epidemiology.

[25] Rahman H., Zafri N.M., Ashik F.R., Waliullah, Khan A. (2021). Identification of risk factors contributing to COVID-19 incidence rates in Bangladesh: A GIS-based spatial modeling approach. Heliyon.

[26] Dowd J.B., Andriano L., Brazel D.M., Rotondi V., Block P., Ding X., Liu Y., Mills M.C. (2020). Demographic science aids in understanding the spread and fatality rates of COVID-19. Proc. Natl. Acad. Sci. USA.

[27] Sannigrahi S., Pilla F., Basu B., Basu A.S., Molter A. (2020). Examining the association between socio-demographic composition and COVID-19 fatalities in the European region using spatial regression approach. Sustain. Cities Soc..

[28] Olds P.K., Musinguzi N., Geisler B.P., Haberer J.E. (2023). Evaluating disparities by social determinants in hospital admission decisions for patients with COVID-19 quaternary hospital early in the pandemic. Medicine.

[29] Assefa Y., Gilks C.F., Reid S., van de Pas R., Gete D.G., Van Damme W. (2022). Analysis of the COVID-19 pandemic: Lessons towards a more effective response to public health emergencies. Glob. Health.

